# Jasmonic Acid Signaling and Molecular Crosstalk with Other Phytohormones

**DOI:** 10.3390/ijms22062914

**Published:** 2021-03-13

**Authors:** Hai Liu, Michael P. Timko

**Affiliations:** Department of Biology, University of Virginia, Charlottesville, VA 22904, USA; hl9h@virginia.edu

**Keywords:** phytohormone, jasmonic acid, JA signaling, crosstalk

## Abstract

Plants continually monitor their innate developmental status and external environment and make adjustments to balance growth, differentiation and stress responses using a complex and highly interconnected regulatory network composed of various signaling molecules and regulatory proteins. Phytohormones are an essential group of signaling molecules that work through a variety of different pathways conferring plasticity to adapt to the everchanging developmental and environmental cues. Of these, jasmonic acid (JA), a lipid-derived molecule, plays an essential function in controlling many different plant developmental and stress responses. In the past decades, significant progress has been made in our understanding of the molecular mechanisms that underlie JA metabolism, perception, signal transduction and its crosstalk with other phytohormone signaling pathways. In this review, we discuss the JA signaling pathways starting from its biosynthesis to JA-responsive gene expression, highlighting recent advances made in defining the key transcription factors and transcriptional regulatory proteins involved. We also discuss the nature and degree of crosstalk between JA and other phytohormone signaling pathways, highlighting recent breakthroughs that broaden our knowledge of the molecular bases underlying JA-regulated processes during plant development and biotic stress responses.

## 1. Introduction

During growth and development, plants are constantly battling against a challenging environment. These adverse or unfavorable environmental conditions are often categorized as: (i) abiotic stresses, such as ultraviolet (UV) radiation, flood, drought, heat, cold, heavy metal toxicity and nutrient deficiency, and (ii) biotic stresses, such as pathogen infection and animal herbivory [1]. Within plant cells, various signal transduction pathways work collaboratively to convey and integrate stress stimuli, and ultimately orchestrate processes of plant growth, development and defense responses [2,3,4]. Phytohormones are among the most important signaling molecules that are involved in the signaling network that regulates these processes [5,6,7,8,9,10,11,12].

Jasmonic acid (JA) and its metabolic derivatives, such as jasmonic acid isoleucine (JA-Ile) and methyl jasmonate (MeJA), collectively known as jasmonates (JAs), are a class of lipid-derived, natural and widely distributed phytohormones in higher plants. JAs have been studied for decades as key signaling compounds involved in many aspects of plant development and stress responses [9,13,14,15,16,17,18]. Upon stress stimuli, such as wounding, herbivory or necrotrophic pathogen infection, plant cells trigger a rapid increase of JAs, which lead to the activation of defense responses and reproduction, as well as the inhibition of growth for plant fitness [19,20,21,22,23]. Moreover, through the crosstalk network, JAs often work in concert with other phytohormones, such as abscisic acid (ABA), auxin, cytokinin (CK), ethylene (ET), gibberellic acid (GA) and salicylic acid (SA), to balance between growth- and defense-related processes, thereby conferring plants acclimation to the changing environments [10,11,24].

Studies in recent decades have remarkably expanded our knowledge on the molecular basis underlying JA biosynthesis, transportation, signal transduction and the crosstalk with other signaling pathways. The history of JA research ever since the first isolation of MeJA in 1962 has been well documented [25]. The importance of JA in many developmental processes, including seedling development, lateral root formation, senescence, flower development, sex determination, and the circadian clock has also been elaborately discussed in several excellent reviews [11,14,17,24,26,27]. In addition, extensive efforts have been made in elucidating the roles JA plays in regulating plant responses to biotic and abiotic stress conditions, as well as the importance of the crosstalk between JA and other phytohormones in these regulations [9,10,16,18,23,28,29,30]. 

In this review, we focus on recent updates on JA biosynthesis and signal transduction mainly in Arabidopsis, the crosstalk complexity between JA and other phytohormone signaling during plant development and stress responses, as well as the roles of the involved transcription factors (TFs) and other regulatory proteins.

## 2. JA Biosynthesis

Thanks to modern technologies and dedicated researchers in biochemistry, cell biology and genetics, the molecular mechanisms underlying JA biosynthesis and signal transduction have been progressively uncovered in both monocotyledon and dicotyledon plants, especially in Arabidopsis [9,14,16,18,22]. Here, we briefly discuss the JA biosynthetic pathway and key enzymes with several highlighted updates.

### 2.1. JA Biosynthesis

**Figure 1:**
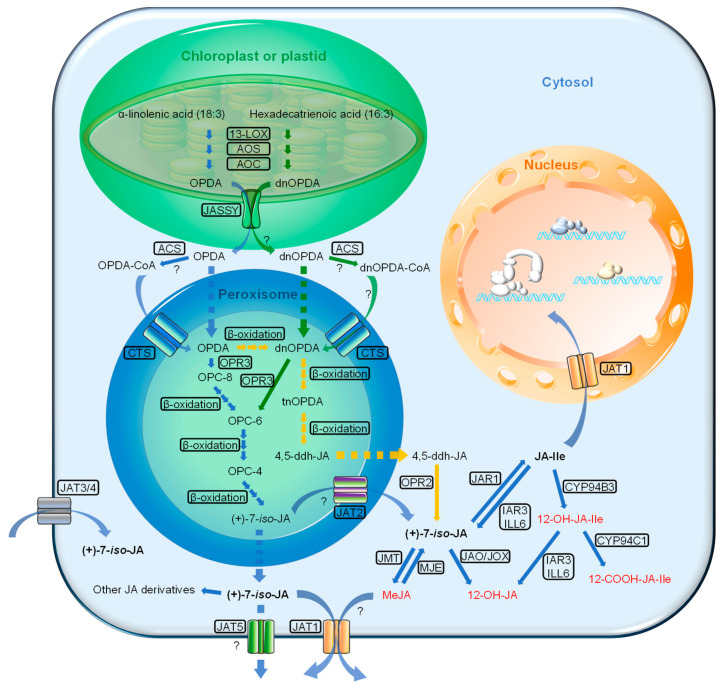
Simplified JA (jasmonic acid) biosynthetic and metabolic pathways and intracellular flux in Arabidopsis. The blue arrows represent the octadecane pathway, the green arrows represent the parallel hexadecane pathway, and the yellow arrows represent the OPR3-independent pathway. Biologically inactive JA derivatives are shown in red. Biosynthetic and metabolic enzymes, as well as transporters are boxed. 13-LOX, 13-lipoxygenase; AOS, allene oxide synthase; AOC, allene oxide cyclase; OPR, OPDA reductase; ACS, acyl-CoA synthetases; JAR1, JA-amido synthetase; IAR3 and ILL6, two JA amidohydrolases; JMT, JA methyl transferase; MJE, MeJA esterase; JAO, JA oxidase; JOX, jasmonate induced oxidase; CYP94B3, JA-Ile-12-hydroxylase; CYP94C1, 12-OH-JA-Ile carboxylase; JASSY, OPDA transporter; CTS, ABC transporter COMATOSE; JAT, jasmonate transporter. dnOPDA, dinor-oxo-phytodienoic acid; tnOPDA, tetranor-OPDA; OPC-8, 8-[3-oxo-2-{pent-2-enyl}cyclopentyl]octanoic acid; OPC-6, 6-[3-oxo-2-{pent-2-enyl}cyclopentyl]hexanoic acid; OPC-4, 4-[3-oxo-2-{pent-2-enyl}cyclopentyl]butanoic acid; 4,5-ddh-JA, 4,5-didehydro-jasmonate; JA-Ile, (+)-7-*iso*-Jasmonoyl-L-isoleucine.

To date, three JA biosynthetic pathways have been identified in Arabidopsis: (1) the octadecane pathway starting from α-linolenic acid (α-LeA, 18:3), (2) the hexadecane pathway starting from hexadecatrienoic acid (16:3), and (3) the 12-oxo-phytodienoic acid (OPDA) reductase 3 (OPR3)-independent pathway (Figure 1). All three pathways require multiple enzymatic reactions that take place sequentially in the chloroplast, peroxisome and finally cytosol. 

The first two pathways start with the release of the polyunsaturated fatty acids α-LeA (18:3) and hexadecatrienoic acid (16:3) hydrolyzed from the membrane of chloroplast or plastid depending on the cell type. Through a sequential series of reactions catalyzed by 13-lipoxygenase (13-LOX), allene oxide synthase (AOS) and allene oxide cyclase (AOC), both the 18:3 and 16:3 are converted to OPDA and dinor-12-oxo-phytodienoic acid (dnOPDA). Then, OPDA is transported from chloroplast into peroxisome, where it gets reduced by OPR3 and subsequently shortened by three rounds of β-oxidation, finally yielding JA [(+)-7-*iso*-JA] (Figure 1). dnOPDA is believed to follow the same pathway as OPDA to produce JA with one less round of β-oxidation [31]. Upon release into the cytosol, JA is then metabolized into a variety of structures through different reactions, such as conjugation with amino acids, hydroxylation, carboxylation and methylation, leading to a collection of JA derivatives with different biological activities [16,22,32]. Among them, the conjugation of JA to the amino acid isoleucine by jasmonoyl-isoleucine synthetase (JAR1) forms the most bioactive form of the hormone, i.e., (+)-7-*iso*-JA-Ile (JA-Ile) [33]. When transferred into the cell nucleus, the bioactive JA-Ile, through a “relief of repression” model, activates several key TFs, such as MYC2, for downstream JA-responsive gene expression [34,35,36,37]. 

The OPR3-independent pathway was recently identified by studying a total loss-of-function OPR3 mutant, *opr3-3* [38]. In the absence of OPR3 activity, OPDA can directly enter the β-oxidation pathway to form dnOPDA, which then gets converted into 4,5-didehydro-JA (4,5-ddh-JA) through two more rounds of β-oxidation. Lastly, 4,5-ddh-JA is reduced to JA by OPR2 in the cytosol (Figure 1). Nevertheless, the majority of JA biosynthesis still occurs through OPR3 [38].

### 2.2. Transporters of JAs and Its Precursors

#### 2.2.1. JASSY

The biosynthesis of JA involves the translocation of the JA-precursor, OPDA, from the chloroplast into the peroxisome. For a long time, the component(s) responsible for the export of OPDA from the chloroplast remained elusive. However, recently, a Bet v1-like family protein, termed JASSY, was identified as the exporter of OPDA from the chloroplast [39]. JASSY is localized to the outer chloroplast envelope where it binds to OPDA and functions as a membrane channel. JASSY loss-of-function mutations in Arabidopsis result in a deficiency in JA accumulation, leading to impairments in pathogen resistance and cold tolerance [39] (Figure 1). Further clarification is needed on whether the chloroplast-derived dnOPDA is also exported by the same means.

#### 2.2.2. Comatose (CTS)

In Arabidopsis, the peroxisomal localized D-type ATP-binding cassette (ABC) transporter CTS (also known as AtABCD1, peroxisomal ABC transporter 1 (AtPXA1), and peroxisomal defective 3 (PED3)) was found to be involved in the peroxisomal import of JA precursors [40]. In addition, the existence of a parallel pathway for passive transport of free OPDA was proposed because both basal level and wound-induced JA synthesis were reduced but not abolished in the *abcd1* mutant Arabidopsis [40]. The identity of the CTS substrates between fatty acids (e.g., OPDA) and fatty acyl-CoA esters (e.g., OPDA-CoA) remained disputable until two groups provided direct evidence that CTS is a transporter of fatty acyl-CoAs and possesses an intrinsic ATP-dependent thioesterase activity, which is essential for the further break down of fatty acids in peroxisome [41,42]. This indicates that OPDA is most likely imported to peroxisome by CTS as a CoA ester (Figure 1). Nevertheless, the cytosolic acyl-CoA synthetase that accounts for the production of OPDA-CoA still remains to be identified. It is also unclear that whether dnOPDA-CoA is formed in cytosol and transported into peroxisome as a substrate of CTS. 

Of note, the fatty acid β-oxidations, which take place exclusively in the peroxisome of plants, contribute to the biosynthesis of not only JA but several other major phytohormones, such as indole-3-acetic acid (IAA) and SA. To our knowledge, CTS/AtABCD1/AtPXA1 is the sole peroxisomal transporter to have been identified that is responsible for the import of, presumably esterified, precursors of JA (OPDA), IAA (indole-3-butyric acid (IBA)) and SA (cinnamic acid (CA)), suggesting a core function of CTS for peroxisome-mediated biosynthesis of phytohormones [43,44,45].

#### 2.2.3. Jasmonate Transporters (JATs) 

Recent studies have found that several members of the G-subfamily of ABC transporters (ABCGs) also function as JA transporters that mediate both intracellular and long-distance JA movement [43,46,47,48,49]. 

A member of the G-subfamily of ABC transporters, AtJAT1/AtABCG16, has recently been identified and characterized as a dual function transporter of JA and JA-Ile in Arabidopsis [47] (Figure 1). AtJA1 is localized at the nuclear envelope and plasma membrane, and respectively mediates the influx of JA-Ile from the cytosol into the nucleus and the cellular efflux of JA to the apoplast. The Arabidopsis loss-of-function mutant *abcg16/jat1* exhibits phenotypes that are consistent with compromised JA signaling [47]. Whether basal diffusion of JA/JA-Ile across the plasma membrane and nuclear envelope takes place awaits further clarification. Nonetheless, AtJAT1 has been shown to be essential in modulating JA-Ile concentration in the nucleus where JA signal perception takes place [47].

More recently, two plasma membrane localized transporters, AtJAT3/AtABCG6 and AtJAT4/AtABCG20, have been identified as potential JA importers that mediate long distance cell–cell translocation of wound-induced JA along the phloem [49] (Figure 1). It has also been demonstrated that AtJAT3 and AtJAT4 work synergistically in core phloem cells with GLUTAMATE RECEPTOR-LIKE3.3 (GLR3.3), an ion channel family member that stimulates distal JA production by transmitting wound-induced calcium (Ca^2+^) fluxes, presumably also through cell–cell transportation. It is hypothesized that AtJAT3- and AtJAT4-mediated loading of locally produced JA drives de novo JA synthesis successively during cell–cell transportation along the phloem passage [49,50,51,52]. Since cell–cell transportation of JA involves both influx and efflux across plasma membranes, it is likely that other JATs, such as the JA exporters AtJAT1 [47] and another potential JA exporter, AtJAT5 [48], are involved. These findings support the idea that JA may act as one component of the mobile molecular signatures in stress- or wound-induced systemic responses [53].

It has been proposed that the peroxisome-localized AtJAT2/AtABCG1 may mediate the peroxisomal export of JA while AtJAT5 mediates the cellular export of JA [48] (Figure 1). As definitive evidence supporting this hypothesis is still lacking, further characterization of JATs is needed to broaden our knowledge on the molecular basis of transporter-mediated distribution and signaling of JA.

Other candidate JA transporters have also been implicated in various studies, including several members of the NITRATE TRANSPORTER1/PEPTIDE TRANSPORTER FAMILY (NPFs) such as NPF2.10/GLUCOSINOLATE TRANSPORTER 1 (GTR1) and NPF4.1/ABA-IMPORTING TRANSPORTER 3 (AIT3) [54,55,56]. However, their functions as JA transporters in planta await further experimental validation [46,48]. Taken together, the exploration of JA biosynthesis, metabolic enzymes and transporters greatly assist our understanding on how plant cells modulate the homeostasis between active and inactive JA components from cytoplasm to nucleus and keep the highly dynamic JA signaling orchestrated in individual cells and throughout the whole plant.

## 3. JA Signaling

### 3.1. JA Perception and Signal Transduction

The generally accepted “relief of repression” model for JA perception is built upon decades of research beginning with the identification of the core co-receptor complex for JA-Ile, that is composed of the F-box protein CORONATINE INSENSITIVE 1 (COI1) containing SKP1-CULLIN1-F-box-type (SCF) E3 ubiquitin ligase complex SCF^COI1^, JASMONATE ZIM DOMAIN (JAZ) proteins and inositol pentakisphosphate (InsP5) [34,35,36,57,58,59,60]. 

**Figure 2:**
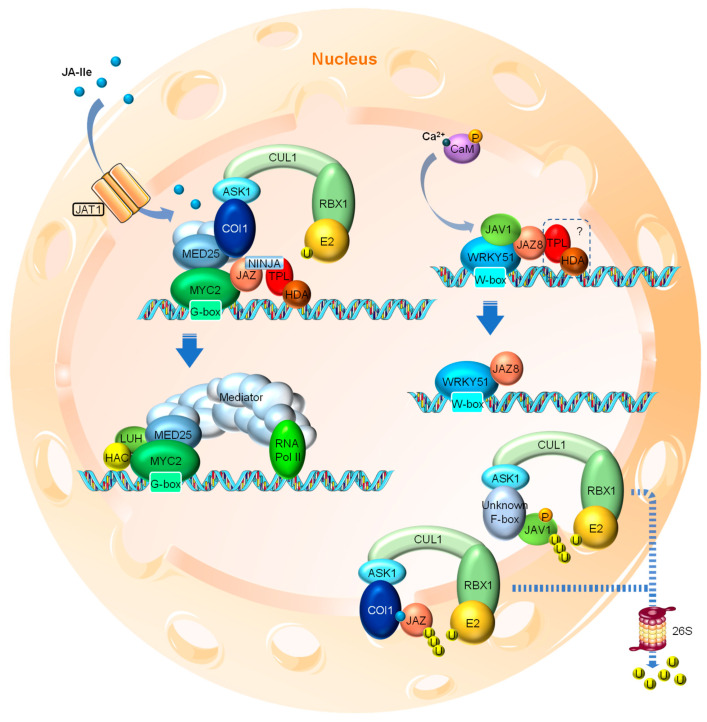
Simplified model of JA signaling in Arabidopsis. When nuclear bioactive JA levels are low, the master transcription factors, such as MYC2, are repressed through the interaction with JAZ proteins that recruit other co-repressors, such as NINJA, TPL and HDA, to form a repressor complex at the promoter regions of JA-responsive genes. In addition, MED25 also physically interacts with MYC2 while bound with COI1, the F-box subunit of the SCF^COI1^ E3 ubiquitin ligase complex. In the case of the JA biosynthesis gene *AOS*, its transcription is repressed by the JJW complex composed of JAV1, JAZ8 and WRKY51. In both cases, the expression of JA-responsive genes is restrained. It is unclear whether the JJW complex also recruits co-repressors, such as TPL and HDA. When a certain developmental or environmental cue triggers the import of bioactive JA (e.g., JA-Ile) into the cell nucleus presumably through the action of JAT1, elevated levels of JA-Ile cause the formation of COI1-JA-JAZ co-receptor complex. The interaction between COI1 and JAZ leads to the dissociation of JAZ and MYC2, as well as the dissociation of COI1 and MED25. As a result, JAZ is degraded via the 26S proteasome and the enhanced interaction between MED25 and MYC2 ultimately leads to MED25-mediated transcriptional activation of the target genes. In the case of JJW-regulated *AOS*, stress-induced fast Ca^2+^ influx leads to the CaM-mediated phosphorylation of JAV1. JAV1 phosphorylation causes the disintegration of the JJW complex and *AOS* transcriptional activation. Phosphorylated JAV1 is subjected to E3 ubiquitin ligase-mediated ubiquitination and 26S proteasomal degradation, although the F-box protein responsible for the specific recognition of JAV1 remains to be identified. JAZ, JASMONATE ZIM DOMAIN; NINJA, NOVEL INTERACTOR OF JAZ; TPL, TOPLESS; HDA, HISTONE DEACETYLASE; MED25, MEDIATOR25; COI1, CORONATINE INSENSITIVE1; ASK1, ARABIDOPSIS SKP1-RELATED1; CUL1, CULLIN1; SCF, SKP1-CULLIN1-F-box; HAC1, HISTONE ACETYLTRANSFERASE1; LUH, LEUNIG_HOMOLOG; JAV1, JASMONATE ASSOCIATED VQ DOMAIN PROTEIN1; AOS, ALLENE OXIDE SYNTHASE; CaM, Calmodulin. RBX1 is a RING finger protein that recruits the E2 ubiquitin-conjugating enzyme to the C-terminus of CUL1.

Under normal conditions, where little or no nuclear JA-Ile is present, certain TFs, such as MYC2 (a basic helix-loop-helix (bHLH) family TF and key activator of JA responses), are repressed by a series of JASMONATE ZIM DOMAIN (JAZ) proteins through direct interaction. MYC2 binds to the G-box motif at the promoter regions of the JA-responsive genes and activates their expression [61] (Figure 2). Most JAZ family members have been shown to interact with MYC2. When binding to MYC2, the JAZ protein recruits the TOPLESS (TPL) and TPL-related (TPR) co-repressors directly or through the adaptor protein NOVEL INTERACTOR OF JAZ (NINJA) to repress the transcriptional activity of MYC2 (Figure 2). The transcriptional repression function of the TPL co-repressors involves the further recruitment of the chromatin modifying HISTONE DEACETYLASE (HDA) complex that “switches off” the targeted region by chromatin condensation [62,63,64]. Members of HDAs such as HDA6 and HDA19 have been shown to participate in JA responses [65,66].

When certain developmental or environmental cues cause a cellular burst of JA, the active derivative JA-Ile is transferred into the nucleus by JAT1 and promotes the formation of the SCF^COI1^-JAZ co-receptor complex, resulting in the ubiquitination of JAZ and its subsequent degradation via the 26S proteasome [13,33,34,35,36,37]. The degradation of JAZ protein thus releases the inhibitory effect on the TFs such as MYC2 (Figure 2), which initiates JA signaling cascades by transcriptional activation of numerous downstream TF genes, such as *ETHYLENE RESPONSE FACTOR1* (*ERF1*) and *OCTADECANOID-RESPONSIVE ARABIDOPSIS59* (*ORA59*), and defense-related responsive genes, such as *VEGETATIVE STORAGE PROTEIN2* (*VSP2*). 

Among the 13 Arabidopsis JAZ proteins identified, five (i.e., JAZ5, JAZ6, JAZ7, JAZ8 and JAZ13) contain the ETHYLENE RESPONSIVE FACTOR-associated amphiphilic repression (EAR) motif responsible for the direct interaction of the protein with TPL co-repressors. Therefore, these JAZs can directly recruit TPLs in the absence of NINJA [67,68]. In addition, a few non-canonical JAZs (such as JAZ8 and JAZ13), which harbor a divergent JA-associated (Jas) domain exhibiting little interaction with COI1, can interact with MYC2 while directly recruiting TPLs through their EAR motifs. Therefore, they are considered as adapters linking TPL to other non-EAR-containing JAZ proteins through heterodimerization [16,67,68,69]. Recently, JAZ8 was shown to form a co-repressor complex with JASMONATE-ASSOCIATED VQ DOMAIN PROTEIN1 (JAV1) and WRKY 51 to bind and repress JA biosynthesis genes, highlighting the role of these non-canonical, EAR motif-containing JAZs [70]. Additionally, a protein named EAR-motif-Containing Adaptor Protein (ECAP) has been reported as a novel adaptor protein that directly interacts with JAZ6 and JAZ8 for the recruitment of the TOPLESS-RELATED2 (TPR2) co-repressor to repress JA responses. Genetic evidence shows that ECAP plays an important part in many JA-regulated processes, including anthocyanin accumulation, JA biosynthesis and defense-related gene activation [71]. Intriguingly, both JAZ6 and JAZ8 harbor an EAR motif which is thought to be adequate for TPL and TPR recruitment. The emerging function of ECAP suggests a novel counterpart of NINJA and a more sophisticated mechanism of gene repression. 

Over the last decade, another key player in JA signaling, MEDIATOR25 (MED25), has been added to the picture [72,73,74]. MED25 is a subunit of the Mediator transcriptional coactivator complex, an evolutionarily conserved multi-subunit complex that plays an essential role in the RNA Polymerase II (Pol II)-dependent transcription throughout eukaryotes [75,76,77]. MED25 physically interacts with COI1 and MYC2, bringing COI1 in close proximity to JAZ, which binds to and represses MYC2 when no or little nuclear JA-Ile is present. Nuclear JA-Ile acts as “molecular glue” to promote the formation of the SCF^COI1^-JAZ co-receptor complex, leading to the weakened interaction between COI1 and MED25, as well as subsequent JAZ degradation. This conformational change also strengthens the interaction between MYC2 and MED25 due to the release of the competitive/interfering effect by JAZ. MED25 then recruits the rest of the Mediator complex and RNA Pol II, as well as other coactivators, such as HISTONE ACETYLTRANSFERASE1 (HAC1) and LEUNIG_HOMOLOG (LUH), to the promoter region of MYC2 target genes for transcriptional activation [74,78,79,80] (Figure 2).

Recently, a WRKY TF and VQ domain protein involved mechanism of herbivory- and wound-induced JA biosynthesis has been reported [70]. The WRKY TFs, a large TF family in plants, have been shown to be crucial in a broad range of developmental and physiological processes, as well as various stress responses [81,82]. The WRKY51 TF forms a heterotrimer complex with JAZ8 and JAV1, a VQ domain protein family member previously identified to specifically modulate JA-regulated plant defense [83]. At resting stage, the JAV1-JAZ8-WRKY51 (JJW) complex represses the expression of JA biosynthesis genes, such as *AOS*, through binding to the W-box motifs in their promoter regions (Figure 2). The herbivory-caused injury rapidly induces cytosolic Ca^2+^ influx, leading to the calmodulin (CaM)-dependent phosphorylation of JAV1 and its subsequent degradation. The disintegration of the JJW complex results in the activation of JA biosynthesis gene expression [70] (Figure 2). Intriguingly, the phosphorylation and degradation of JAV1 triggered by Ca^2+^/CaM is independent of JA-Ile elicitation [70], which seems in contrast to earlier data suggesting that JA triggers the degradation of JAV1 in a COI1-dependent manner [83]. Resolving these conflicting findings clearly needs further exploration. It also remains to be determined whether the JJW-directed mechanism is acting in parallel to the canonical JAZ-regulated mechanisms (e.g., JAZ-MYC) and whether inputs from different upstream signals (Ca^2+^ vs. JA-Ile) can be simultaneously perceived by the promoters of JA biosynthesis genes (e.g., *AOS*) that are also directly regulated by MYC2 [84]. Finally, whether MED25 is involved in the JJW regulation needs additional confirmation. Regardless, this finding is undoubtedly a milestone that adds another layer of transcriptional regulation to wound-induced JA biosynthesis, highlighting both the regulatory mechanism of JA biosynthesis and the function of the non-canonical JAZ proteins like JAZ8. As discussed before, JAZ8 is believed to be resistant to COI1-mediated degradation due to its divergent Jas domain. Therefore, no JAZ8 degradation has been reported by Yan et al. [70]. JAZ8 now has exhibited interaction with not only MYC2 but also WRKY51, it is highly likely that JAZ8 also recruits TPL and HDA co-repressors to the JJW complex since the repression of the JJW complex largely depends on the EAR motif of JAZ8.

Other evidence also suggests that WRKY33 and WRKY57 interact with two VQ proteins SIGMA FACTOR BINDING PROTEIN1 (SIB1) and SIB2 while potentially being able to interact with JAZ4 and JAZ8, although further study is needed to elucidate the details of the molecular context [85,86].

### 3.2. JA-Regulated Transcription Factors

**Figure 3:**
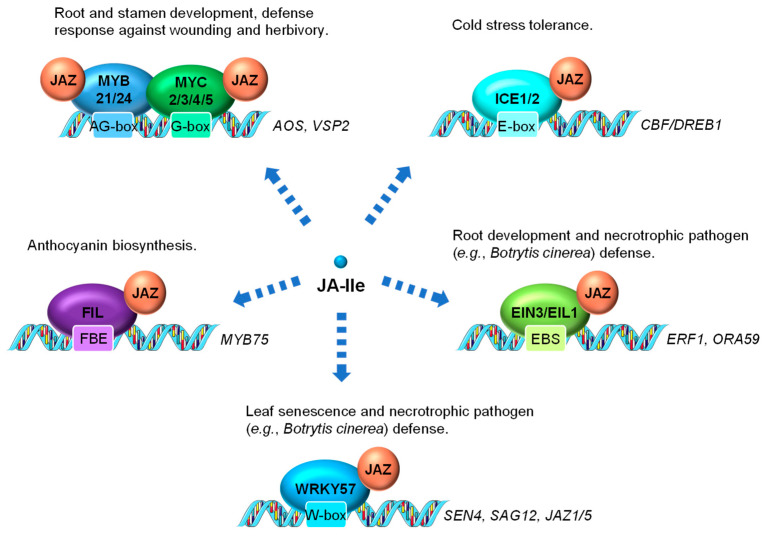
Examples of JA-regulated processes control through the interaction between JAZ and transcription factors (TFs). The MYC TFs, several R2R3-MYB family members (including MYB21 and MYB24), and other TFs (e.g., ICE1, EIN3, EIL1, and FIL) are direct targets of JAZ repressors. These TFs are activated by JA-mediated JAZ degradation and positively regulate JA responses. WRKY57 is also a direct target of JAZ but acts as a negative regulator of JA regulated leaf senescence and defense against necrotrophs. ICE1, INDUCER OF CBF EXPRESSION1; EIN3, ETHYLENE INSENSITIVE3; EIL1, EIN3-LIKE1; FIL, FILAMENTOUS FLOWER; EBS, EIN3 binding site; FBE, FIL DNA binding element.

In addition to MYC2 serving as the main transcriptional regulator of JA-induced gene activation, other members of the MYC TF family as well as members of other TF families have also been shown to be directly involved in controlling JA-regulated gene expression (Figure 3). MYC3 and MYC4 are also targets of JAZ repressors (e.g., JAZ3 and JAZ5) and act additively with MYC2 to activate JA response in the vegetative tissue, especially the JA-dependent defense response against wounding and herbivory [87,88]. MYC2, MYC3, MYC4 and MYC5 interact with at least two R2R3-MYB TFs, MYB21 and MYB24, to form a MYC-MYB transcription complex (Figure 3). Both MYC and MYB are repressed by JAZ suppressor and are activated by JA to cooperatively regulate stamen development in Arabidopsis [89,90,91,92]. In rice, data have also shown that the JA-responsive R2R3-type MYB TFs, JAMYB and its homolog, are transcription activators directly regulated by JA [93]. JAMYB binds to the AG-motif-like motif in the promoter region of *Argonaute18* (*AGO18*) gene, which encodes a core RNA silencing component that promotes AGO1-mediated antiviral RNAi [94]. The transactivation activity of JAMYB is normally repressed by JAZ6. The JA accumulation elicited by rice stripe virus coat protein triggers the ubiquitination and proteasomal degradation of JAZ6, relieving the repression of JAMYB to activate the expression of *AGO18*. Elevated accumulation of AGO18 ultimately leads to enhanced antiviral defense in rice [93,94]. It is reasonable to hypothesize that certain rice MYC homologs also interact with JAMYB. 

INDUCER OF CBF EXPRESSION1 (ICE1) and ICE2 are two MYC-like bHLH transcriptional activators playing critical roles in modulating cold stress responses [1,95]. Both are repressed by JAZ repressors (e.g., JAZ1 and JAZ4) through physical interaction [96,97] (Figure 3). Cold stress-induced endogenous JA production triggers the turnover of JAZ repressors and the activation of ICE1 and ICE2, which further activate the C-REPEAT BINDING FACTOR/DRE BINDING FACTOR1 (CBF/DREB1) transcriptional cascade for cold stress tolerance [96,97]. 

Several members in the APETALA2/ETHYLENE RESPONSE FACTOR (AP2/ERF) TF family, such as ETHYLENE INSENSITIVE3 (EIN3), EIN3-LIKE 1 (EIL1), ERF1 and ORA59 comprise the classical ERF branch of JA signaling, which is marked by the activation of the downstream defense gene *PLANT DEFENSIN1.2* (*PDF1.2*) [98]. Transcription of ERF1 and ORA59 is directly controlled by EIN3 and its homolog EIL1, which are identified as direct targets of JAZ proteins and activated by JA (Figure 3). EIN3 and EIL1 have been shown to play important roles in mediating JA-induced processes, such as root development and defense responses to necrotrophic fungi [66]. EIN3 and EIL1 are two well-recognized TFs essential for the activation of ethylene (ET) responses [99] and thus are likely to be key components necessary for integrating JA and ET signaling. 

WRKY57, a WRKY TF involved in both JA-induced leaf senescence and necrotrophic pathogen response in Arabidopsis, is repressed by JAZ4 and JAZ8 through physical interaction [85,86]. WRKY57 directly binds to the promoters of *SENESCENCE4* (*SEN4*) and *SENESCENCE-ASSOCIATED GENE12* (*SAG12*), as well as another two JAZ repressor genes (*JAZ1* and *JAZ5*) to regulate their expression (Figure 3). Intriguingly, genetic evidence shows that the loss-of-function of *WRKY57* enhances JA-mediated leaf senescence and resistance against *Botrytis cinerea* (*B. cinerea*) infection, suggesting it a negative regulator of JA signaling [85,86].

A YABBY (YAB) TF family member FILAMENTOUS FLOWER (FIL)/YAB1 has been found as a direct target of JAZ3 (Figure 3). Via JA-triggered degradation of JAZ3, FIL/YAB1 promotes anthocyanin biosynthesis through, at least in part, direct transcriptional activation of MYB75 [100], a key component of the WD-repeat/bHLH/MYB transcription complex that is also repressed by several JAZ repressors [101,102].

Whether these JAZ-regulated TFs also share other components of the regulatory mechanism (e.g., MED25, TPL, HAD, and HAC), and if they act synergistically or independently in response to different types of stimuli upstream of JA, are certainly questions for future research.

### 3.3. Negative Feedbacks and Termination of JA Signal

Since JA is a stress signal that generally leads to growth inhibition, proper desensitization and termination of the JA signal is undoubtedly as important as its activation for overall plant growth and fitness. In fact, the JA signal is elaborately controlled at multiple levels to ensure that each response only lasts for an appropriate period at an appropriate amplitude [30,80,103]. 

Cytosolic JA-Ile dynamics are shaped by at least two JA-inducible catabolic pathways in Arabidopsis. The first pathway is the direct oxidation of JA-Ile by members of the cytochrome P450 subfamily 94 (CYP94) enzymes, CYP94B1, CYP94B3 and CYP94C1, which turn JA-Ile into bio-inactive 12OH-JA-Ile and 12COOH-JA-Ile [104,105] (Figure 1). The second pathway is the deconjugation of JA-Ile mediated by two amidohydrolases, IAR3 and ILL6, which hydrolyze both JA-Ile and 12OH-JA-Ile [106] (Figure 1). Both pathways have been shown to contribute additively for the turnover of JA-Ile but act differently for JA responses and tolerance to related stress conditions [103,107]. In crop plants (e.g., rice and corn), JA catabolism has also been proven to be crucial to both the development, such as sexual determination [108,109], and stress tolerances, such as salt and cold [110,111]. 

JA-Ile stimulates rapid activation of *JAZ* gene expression while most *JAZ* genes in Arabidopsis can produce truncated JAZ splice variants that can still bind to the MYC proteins but have little capability of forming complexes with JA-Ile and COI1 for proteasomal degradation. Overexpression of certain JAZ splice variants, such as JAZ10, result in dominant repression of JA responses [112,113]. Crystal structure reveals that the JAZ10 splice variant tightly binds to MYC3 and blocks the interaction between MYC3 and MED25, which is crucial for the transcriptional activation of MYC3 target genes [114]. These findings indicate that the alternative splicing of JAZ genes serve as a general feedback mechanism to desensitize JA signaling. Intriguingly, JA also induces the recruitment of two splicing factors, PRE-mRNA-PROCESSING PROTEIN 39a (PRP39a) and PRP40a to *JAZ* loci by MED25. PRP39a and PRP40a, in turn, facilitate the full splicing of *JAZ* transcripts to produce the full-length JAZ proteins, thus preventing the excessive desensitization of JA responses caused by the overaccumulation of JAZ splice variants [115]. These data suggest that the JA-induced negative feedback mechanism by the alternative splicing of *JAZ* genes is under exquisite modulation.

Several bHLH family subgroup IIId members (e.g., the JASMONATE-ASSOCIATED MYC2-LIKE proteins (JAMs) in Arabidopsis and the MYC2-TARGETED BHLHs (MTBs) in tomato) have been identified as negative regulators of JA responses [116,117,118,119]. In Arabidopsis, JAM1/bHLH17, JAM2/bHLH13 and JAM3/bHLH3 interact with JAZs and function as transcriptional repressors by competing with MYCs for G-box binding [116,117,118]. Likewise, the tomato MTB1, MTB2 and MTB3 are activated by MYC2 but act in turn to negatively regulate JA responses by competing with MYC2 for the target promoter binding site (i.e., the G-box motif), impeding the formation of the MYC2-MED25 complex [119]. In addition to the activation of JAMs or MTBs, JA also stabilizes BTB/POZ-MATH3 (BPM3), one of the BPM proteins that function as adaptors of Cullin3-based E3 ubiquitin ligases [120]. Several BPMs are found to directly target MYC2, MYC3 and MYC4 for polyubiquitination and degradation. Thus, the stabilities of JA-activated MYCs are negatively controlled by BPMs, especially BPM3, whose stability is greatly enhanced by JA [120]. 

Taken together, these groundbreaking discoveries suggest that plant cells orchestrate a complex and autoregulatory negative feedback circuit to desensitize and terminate JA signals at multiple layers. 

## 4. Crosstalk Complexity of JA with Other Phytohormones

Data accumulated over years of research have revealed that several signaling pathways, including JA, auxin and GA, share a highly conserved mechanistic framework for gene regulation. The SCF^F-box^ E3 ligase complex-mediated turnover of the repressive proteins to activate the master TFs appears to be a universal mechanism among several phytohormones [121]. For instance, parallel to JA signaling, in which JAZ repressors are degraded through SCF^COI1^ receptor recognition to release the repression on MYC TFs, the auxin-induced activation of auxin-responsive genes is achieved through the degradation of AUXIN/INDOLE-3-ACETIC ACID (Aux/IAA) repressors that interact with AUXIN RESPONSE FACTOR (ARF) transcription activators. The degradation of Aux/IAA repressors is mediated by the SCF^TRANSPORT INHIBITOR RESPONSE1 (TIR1)^ receptor complex [122,123]. In addition, the DELLA proteins, master negative regulators of GA signaling, are subject to SCF^SLEEPY1 (SLY1)^-mediated degradation in response to GA [124]. Ultimately, the combinational alteration of the regulatory proteins at the promoter region leads to the change of the target gene status (inactive or active) at the chromatin level. Modern evidence has linked many enzymes involved in epigenetic modifications (e.g., HDAs and HACs) to phytohormone-mediated gene regulations [63,64,125]. 

Although participating in many developmental processes, JA is generally recognized as a stress hormone in response to various biotic and abiotic stresses. Within plants, numerous signal transduction pathways intertwine and interact in a complicated but highly arranged manner in response to stress(es) [126]. Although only the tip of the iceberg has been discovered so far to understand how many and how these pathways influence one another from the initial onset to the mobilization of target regulon and finally the cease of signals at a given stress or various combinations of stresses, numerous molecules such as receptors, kinases, TFs and other small regulatory proteins have been identified and characterized to mediate the JA signaling pathway and its crosstalk with other phytohormone signaling pathways [10,18,23,127]. It is believed that these TFs, such as bHLH, MYB, ERF and WRKY TFs, and regulatory co-factors, such as JAZ, DELLA and AUX/IAA proteins, hold the keys to the intricate crosstalk among different signaling pathways.

### 4.1. JAZ vs. DELLA

**Figure 4:**
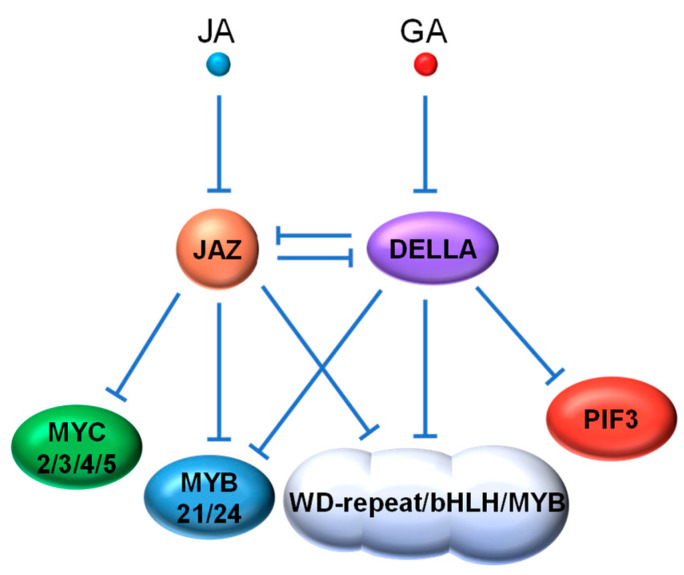
Simplified schematic of crosstalk between JA and GA signaling pathways. Both the antagonism and synergy between JA and GA signaling pathways are mainly mediated by the crosstalk between JAZ and DELLA proteins. Through binding competition, JAZ and DELLA reciprocally affect each other’s ability or availability to repress their respective target TFs (i.e., MYC2 for JA signaling; PIF3 for GA signaling). Both DELLAs and JAZs also interact with the bHLH and MYB components in the WD-repeat/bHLH/MYB and MYC-MYB complexes to repress their transcriptional activity. JA and GA, respectively, induce the degradation of JAZs and DELLAs, which additively activate JA- and GA-mediated processes, such as trichome formation and filament elongation. PIFs, phytochrome interacting factors.

The antagonism between two phytohormones often involves the enhanced repression of one hormone caused by another. Examples of this antagonism can be seen in the crosstalk between JA and GA, two major phytohormones responsible for defense responses and growth processes, respectively. The crosstalk between the JA and GA pathways occurs partially through the interaction between their key repressors, the JAZ and DELLA proteins [128,129]. Similar to JAZ repressors, Arabidopsis DELLA proteins, such as GIBBERELLIC ACID INSENSITIVE (GAI), REPRESSOR OF GA (RGA), RGA-like1 (RGL1), RGL2 and RGL3, act as negative regulators of their target TFs (e.g., phytochrome interacting factors (PIFs)) through direct interaction to suppress related GA responses [130]. It was first discovered that DELLA proteins can compete with MYC2 for binding to JAZ1 and thus relieve MYC2 in a certain degree for JA signal activation (Figure 4). An elevated GA level triggers the degradation of DELLAs, freeing up JAZ1 for enhanced repression of MYC2 to attenuate JA signaling [131]. Reciprocally, JA increases the stability of DELLA proteins (e.g., RGA), presumably through JAZ degradation, thereby improving the repression of GA-related TFs, such as PIF3 [132]. A recent study in rice also demonstrated that the interaction between OsJAZ9 protein and the DELLA protein SLENDER RICE 1 (SLR1) mediates the antagonism between JA and GA [133].

Interestingly, synergy between JA and GA has also been observed in some defense response. The WD-repeat/bHLH/MYB TF complexes are key controllers of anthocyanin biosynthesis and trichome formation, that contribute significantly to insect resistance in plants [134,135]. The bHLH components (i.e., Glabra3 (GL3), Enhancer of Glabra3 (EGL3) and Transparent Testa8 (TT8)) and the R2R3 MYB components (i.e., MYB75 and Glabra1 (GL1)) have been identified as targets of both JAZ and DELLA repressors [101,102,136,137]. Both DELLAs and JAZs interact with the WD-repeat/bHLH/MYB complex to repress its transcriptional activity. JA and GA respectively induce the degradation of JAZs and DELLAs, which additively activate the WD-repeat/bHLH/MYB complex for trichome formation. Further evidence for JA and GA synergy comes from the involvement of DELLAs in previously discovered MYC-MYB regulation by JA that modulates late stamen development [89,90,91]. Huang et al. [138] demonstrated that DELLAs directly interact with MYB21 and MYB24, the MYB components of the MYC-MYB complex, and that JAZs and DELLAs act coordinately to inhibit MYB function that is needed for filament elongation (Figure 4). 

### 4.2. JA vs. Auxin

**Figure 5:**
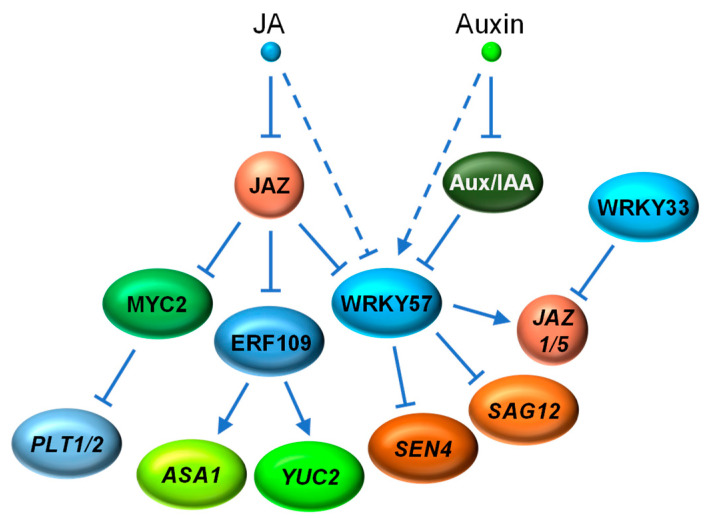
Simplified schematic of crosstalk between JA and auxin signaling pathways. JA activates ERF109 which further activates the expression of *ASA1* and *YUC2* for auxin production that is required lateral root formation. JA-activated MYC2 directly represses the expression of *PLT1* and *PLT2* to inhibit primary root growth. As a negative regulator of JA-mediated leaf senescence and necrotrophic pathogen defense, the WRKY57 TF represses the expression of *SEN4* and *SAG12* while activating the expression of *JAZ1* and *JAZ5*. Members of both JAZ (JAZ4 and JAZ8) and Aux/IAA (IAA29) families competitively interact with WRKY57. The protein level of WRKY57 is negatively regulated by JA but positively regulated by auxin. *ASA1*, *ANTHRANILATE SYNTHASE α1*; *YUC2*, *YUCCA2*; *PLT*, *PLETHORA*; *SEN4*, *SENESCENCE4*; *SAG12*, *SENESCENCE*-*ASSOCIATED GENE12*; Aux/IAA, AUXIN/INDOLE-3-ACETIC ACID.

The interplay between JA and auxin signaling is also essential for plant development. It has been reported that JA induces lateral root formation by promoting auxin biosynthesis [139]. Further studies revealed that the wound-induced JA activates ERF109 by removing the JAZ repression. ERF109 binds to the GCC-box element in the promoters of *ANTHRANILATE SYNTHASE α1* (*ASA1*) and *YUCCA2* (*YUC2*), two genes encoding key enzymes in auxin biosynthesis, and promote their expression for auxin production required for lateral root formation [27,139,140] (Figure 5). 

In contrast to its canonical role in activation, JA-activated MYC2 directly represses the expression of *PLETHORA1* (*PLT1*) and *PLT2*, two AP2-domain TF genes that are essential for auxin-induced root stem cell niche patterning [141]. Therefore, the MYC2-mediated repression of PLT1 and PLT2 contributes, at least partly, to the inhibition of primary root growth by JA [141] (Figure 5). 

The Arabidopsis WRKY57 also exhibits bifunctional attributes as a negative regulator of both leaf senescence and necrotrophic pathogen defense [85,86]. During JA-induced leaf senescence, JA triggers the degradation of WRKY57 to activate the expression of *SEN4* and *SAG12*, two senescence-associated genes normally repressed directly by WRKY57. Consistent with the antagonism between JA and auxin, auxin increases the protein level of WRKY57. Furthermore, both JAZ (JAZ4 and JAZ8) and Aux/IAA (IAA29) proteins competitively interact with WRKY57, suggesting that WRKY57 serves as a point of converging for JA and auxin signaling in leaf senescence [85]. Subsequently, it was discovered that WRKY57, instead of repressing gene expression, activates the expression of two JA repressor genes, *JAZ1* and *JAZ5*, to negatively regulate plant defense against necrotrophic pathogen *B. cinerea* [86]. Interestingly, *JAZ1* and *JAZ5* are direct targets of both WRKY33 and WRKY57. WRKY33, acting opposite to WRKY57, positively regulates plant necrotrophic resistance by transcriptional repression of JAZ1 and JAZ5 [86,142,143] (Figure 5).

### 4.3. JA vs. ET 

**Figure 6:**
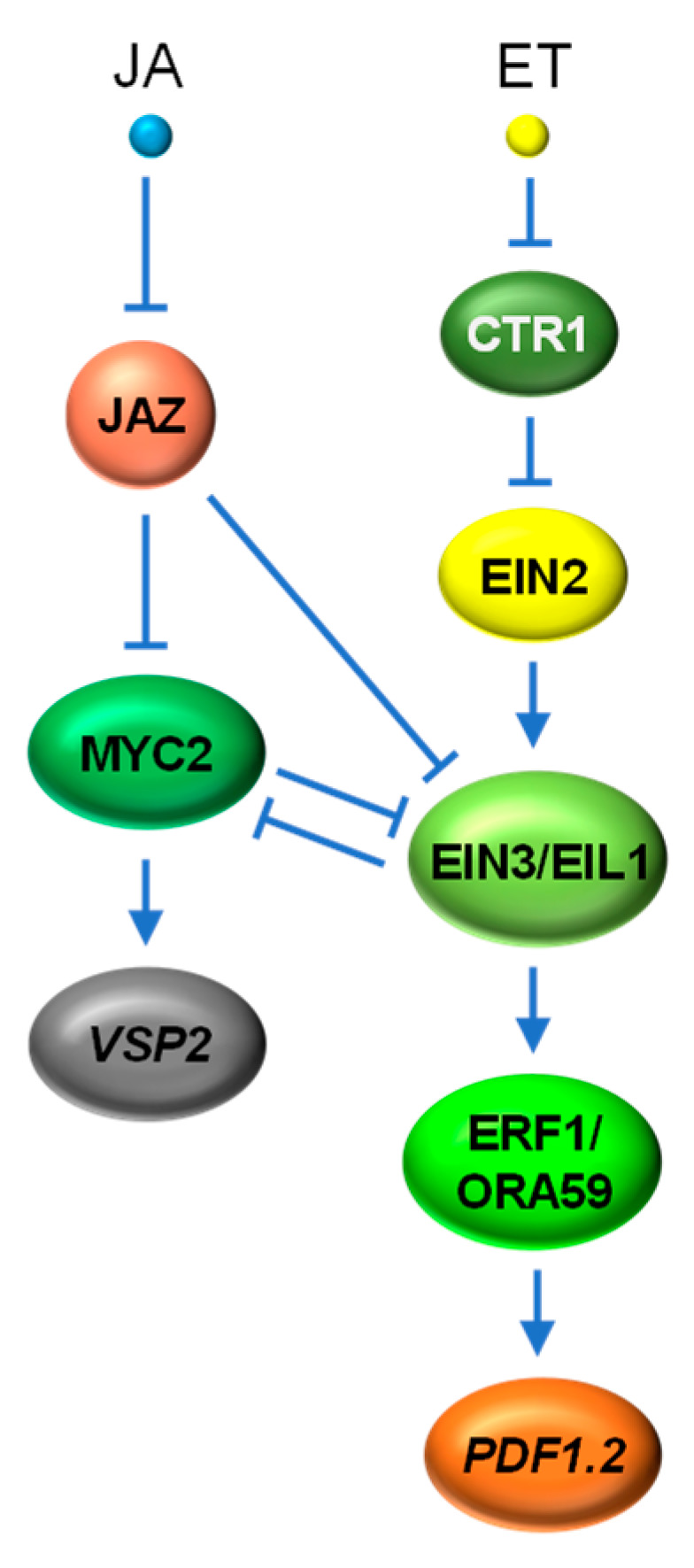
Simplified schematic of crosstalk between JA and ET signaling pathways. JA activates MYC2 to initiate wound and herbivory responses through the transactivation of defense genes, such as *VSP2*. JA-mediated JAZ degradation also de-represses EIN3 and EIL1, the two master TFs of ET signaling, for the expression of other ERF TFs, such as ERF1 and ORA59, targets of which include pathogen defense genes, such as *PDF1.2*. Additionally, MYC2 and EIN3 physically interact with each other and mutually inhibit each other’s transcriptional activities to balance between development and different defense responses. *VSP2*, *VEGETATIVE STORAGE PROTEIN2*; *PDF1.2*, *PLANT DEFENSIN1.2*; ERF1, ETHYLENE RESPONSE FACTOR1; ORA59, OCTADECANOID-RESPONSIVE ARABIDOPSIS59; EIN, ETHYLENE INSENSITIVE; EIL1, EIN3-LIKE1; CTR1, CONSTITUTIVE TRIPLE RESPONSE1.

ET is another major phytohormone hallmarked as a regulator of plant development and defense against necrotrophic fungi. A large number of studies have shown that JA and ET act synergistically in plant defense responses through the activation of several AP2/ERF TFs, such as ERF1, ERF2, ERF14 and ORA59, which then transactivate a set of defense-related genes, including *PDF1.2*, *BASIC CHITINASE* (*ChiB*) and *AGMATINE COUMARYL TRANSFERASE* (*ACT*) [144,145,146,147]. The molecular evidence directly linking JA and ET signaling pathways came from the characterization of two Arabidopsis ET-stabilized TFs, EIN3 and EIL1 [66]. EIN3 and EIL1 act upstream of ERFs, including ERF1 and ORA59, and are considered as key transcriptional regulators of ET responses through the cascadic activation of ethylene-responsive genes, such as *PDF1.2* [99,148]. Zhu et al. [66] demonstrated that JAZs repress the function of EIN3 and EIL1 through physical interaction. Such repression is relieved by JA-induced turnover of JAZ repressors, leading to the enhanced expression of ET-responsive genes mediated by EIN3 and EIL1 (Figure 6). 

Recent studies have shown that MYC TFs can inhibit ERF TFs within the JA signaling pathway to antagonize ET signaling. MYC2 and EIN3, the two master TFs of JA and ET signaling pathways, were also found to physically interact with each other and mutually inhibit each other’s transcriptional activity to coordinate plant development and defense responses [149,150,151] (Figure 6). Nevertheless, the exact molecular basis on how MYC2 and EIN3/EIL1 interfere or coordinate with each other for the expression of their respective regulon under various developmental and stress scenarios remains to be clarified.

### 4.4. JA-ABA

The phytohormone ABA is well documented in its ability to mediate developmental processes and abiotic stress tolerance, particularly drought and salinity stress [152,153]. ABA binds to members of the PYRABACTIN RESISTANCE/REGULATORY COMPONENT OF ABSCISIC ACID RECEPTOR (PYL/RCAR) ABA receptor family to initiate signal transduction. Upon ABA binding, these receptors form a stable complex with type 2C protein phosphatases (PP2Cs), leading to the release of SNF1-related kinases 2 (SnRK2s) that are normally bound and suppressed by PP2Cs. Subsequently, the activated SnRK2s activate their downstream TFs, such as the ABSCISIC ACID-INSENSITIVE5 (ABI5) and the ABA-RESPONSIVE ELEMENT BINDING PROTEIN/ABRE-BINDING FACTOR (AREB/ABF) basic-domain leucine zipper (bZIP) TFs, by phosphorylation to mediate ABA signaling [153,154].

**Figure 7:**
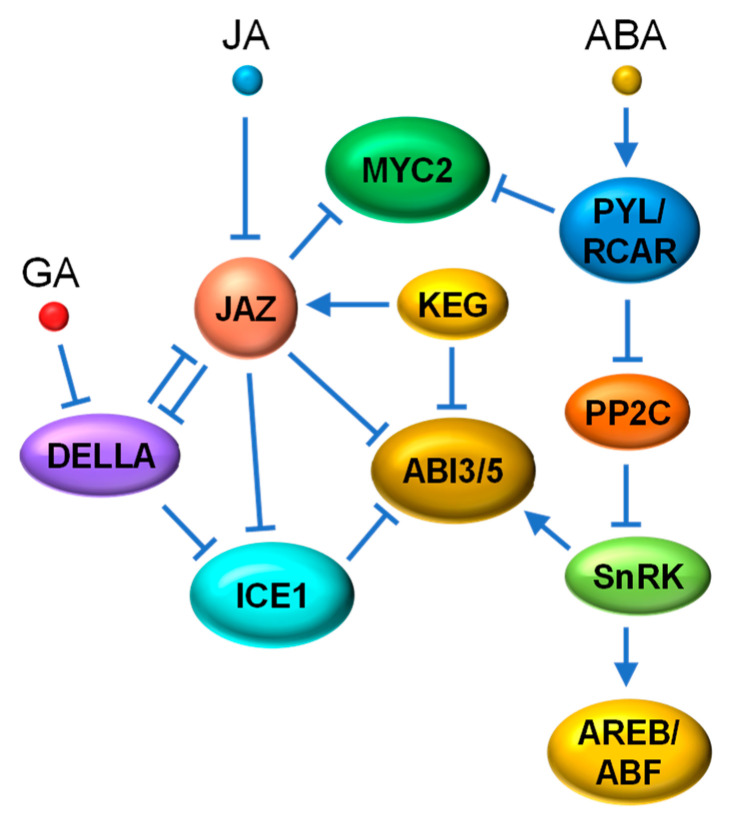
Simplified schematic of crosstalk between JA and ABA signaling pathways. The interaction between JAZ and ABI3 and ABI5 plays an important part in JA and ABA synergy. JA-mediated JAZ degradation releases ABI3 and ABI5, the positive TFs of ABA signaling. ABA also induces JA biosynthesis, which is thought to be mediated by the ABA-activated AREB/ABF TF members based on a recent study in rice. Antagonistically, the ABA receptor PYL6 interacts with MYC2 and negatively regulates its activity in an ABA-dependent manner. KEG acts as a negative regulator of both JA and ABA signaling by stabilizing JAZ12 and triggering ABI5 degradation. The transcriptional activity of ABI5 is also repressed through physical interaction with ICE1. ICE1, as a negative regulator of ABA signaling, is repressed by DELLA proteins of the GA signaling pathway. ABI5, ICE1, and DELLA proteins are also targets of JAZ proteins. PYL/RCAR, PYRABACTIN RESISTANCE/REGULATORY COMPONENT OF ABSCISIC ACID RECEPTOR; PP2C, type 2C protein phosphatase; SnRK2, SNF1-related kinases2; ABI5, ABSCISIC ACID-INSENSITIVE5; AREB/ABF, ABA-RESPONSIVE ELEMENT BINDING PROTEIN/ABRE-BINDING FACTOR; KEG, KEEP ON GOING.

In general, ABA and JA act synergistically in processes such as seed germination inhibition and herbivory defense, and antagonistically in several developmental processes [10]. Early molecular studies provided evidence linking core ABA signaling to JA signaling by the identification of the JA-induced ABA receptor PYL4 [155]. Two ABA receptor mutants, *pyl4* and *pyl5*, exhibit JA-hypersensitive shoot growth and reduced anthocyanin accumulation in response to JA [155]. In addition, a direct interaction between MYC2 and another ABA receptor PYL6 was reported and it was discovered that PYL6 negatively regulates MYC2 activity in an ABA-dependent manner [156] (Figure 7). These data suggest that JA signaling is dependent on ABA. The recent discovery of chloroplast-localized PLASTID LIPASE2 (PLIP2) and PLIP3, two ABA-induced phospholipase A that are involved in the biosynthesis of JA and related oxylipins, provides a probable mechanistic link between ABA-dependent JA accumulation and their synergistic abiotic stress responses [157]. More recently, the identification of the “OSMOTIC STRESS/ABA-ACTIVATED PROTEIN KINASE10 (SAPK10)-bZIP72-*AOC*” pathway in rice has provided another clue to the ABA-promoted JA biosynthesis that results in the synergistical inhibition of seed germination [158]. SAPK10, a rice SnRK2 member that is involved in the activation of ABA signaling, is able to activate and stabilize the bZIP TF, bZIP72, through direct phosphorylation. bZIP72 then activates *AOC* expression by binding to the G-box element in *AOC* promoter, thus elevating the endogenous level of JA [158].

The RING-type ubiquitin E3 ligase KEEP ON GOING (KEG) functions as a negative regulator of ABA signaling by directly binding to ABI5, leading to its ubiquitination and degradation. KEG has also been shown to interact with JAZ12 and enhances its stability by interfering with its JA-triggered degradation [159]. Therefore, KEG, as a negative regulator of both JA and ABA signaling, appears to play a specific role in the crosstalk between JA and ABA. Interestingly, a subset of JAZ repressors, including JAZ3, interact with ABI5 and suppress its transcriptional activity. ABA-induced biosynthesis of JA can release ABI5 to activate the expression of ABA-responsive genes through JAZ degradation [160]. A more recent study proved that the interaction between JAZ and ABI3 and ABI5 serves as an essential molecular basis underlying the JA-induced activation of ABA signaling [161] (Figure 7). Evidence shows that exogenous JA triggers COI1-mediated JAZ degradation to release ABI3 and ABI5, positive TFs of ABA signaling, leading to the enhanced inhibition of seed germination [161]. 

It is well known that GA promotes seed germination by counteracting ABA action. This has been elucidated at the molecular level by experimental demonstrations that connect DELLA repressors to ABI5 [162]. For example, DELLA proteins physically interact with and repress the regulatory effect of ICE1. ICE1 is a negative regulator of ABA signaling that impairs the transcriptional activity of ABI5 through physical interaction and directly repressing the expression of several ABA responsive genes [162]. By triggering the degradation of DELLAs, GA suppresses ABI5-mediated ABA signaling via stabilized ICE1. Most interestingly, DELLAs [131,133], ICE1 [96] and ABI5 [160,161] are all targets of JAZ proteins (Figure 7). Therefore, ABI5 appears to be the convergence point where ABA signaling is fine-tuned through the interaction of regulators coming from multiple signaling pathways (such as JA and cold). 

Additionally, the bHLH TF ABA-INDUCIBLE BHLH-TYPE TRANSCRIPTION FACTOR (AIB)/JAM1, previously reported as an ABA-induced positive regulator of ABA signaling [163], was demonstrated as a negative regulator in JA signaling, indicating that JAM1 underlies the antagonism between in JA and ABA signaling [164]. The AP2/ERF TF ORA47 was shown to regulate the expression of multiple biosynthetic and/or signaling genes for both JA (e.g., *DAD1*, *AOC1* and *MYC2*) and ABA (e.g., *ABI2*, *NINE-CIS-EPOXYCAROTENOID DIOXYGENASE3* (*NCED3*) and *NCED9*) through binding to their promoters. Overexpression of *ORA47* significantly increased ABA and JA accumulation in plants under stress conditions [165]. By closely examining the data on repeated dehydration stress in plants, Avramova [166] suggests that the crosstalk between JA and ABA signaling pathways is driven by a memorable, highly dynamic and self-reprogrammable regulatory mechanism, which supports the general observation that ABA and JA function both synergistically and antagonistically depending on the combination of innate growing stage, developmental status and stress(es).

In addition to the crosstalk mentioned above, JA signaling also actively interacts with other hormone signaling pathways, such as cytokinin, brassinosteroid (BR) and SA [11,129,167,168,169]. JA has also been reported to be involved in a wide range of abiotic stress responses, including salt, drought, heavy metal, high and low temperature, light stress and ozone stress [23,127]. Furthermore, mounting evidence suggests that JA and its precursors, such as OPDA, participate in systemic signaling in plants, through which local stress can be perceived throughout the entire plant to induce systemic defense response or systemic acquired acclimation (SAA) [47,49,53,170,171,172,173]. 

## 5. Future Perspectives

We have just now begun to uncover the functionality and significance of JA signaling that is indispensable for plant development and stress tolerance. It remains to be determined whether different environmental cues trigger different compositions of systemic signals and whether different forms of JA and its precursors are differentially transmitted as different environmental signals. Why there are a plethora of JA derivatives is still unknown, as is whether these different forms of JA have other functions yet to be discovered. It also is unknown how bioactive JA is purged from the cell nucleus when the level of signal needs to be dampened or JA signaling is no longer needed.

Plants have evolved sophisticated and efficient perception, signaling and regulatory networks to survive different stress conditions at the cost of reduced growth and yield. Therefore, fully understanding the integrated molecular mechanisms, including the hormone signaling crosstalk, at the genome-scale will greatly help increase plant resilience towards the changing global climate and further assist in the improvement of horticultural and agricultural productivity. Although the history of plant research has greatly increased our knowledge on how plants achieve their ultimate fitness through the implementation and coordination of different signaling pathways at a given developmental stage and in response to various abiotic and biotic stress conditions, it still remains a challenge to elucidate the dynamic interactions among various signaling pathways that occur during the life cycle of a plant under static environments and in the changing environments encountered in nature. This partially explains why only a small fraction of academic research discoveries are translated to and adopted as field applications. Nevertheless, science-based agricultural improvements continue to provide important advancements that benefit humankind today.

## Data Availability

No new data were created or analyzed in this study. Data sharing is not applicable to this article.
